# A scalable synthesis of the (*S*)-4-(*tert*-butyl)-2-(pyridin-2-yl)-4,5-dihydrooxazole ((*S*)-*t-*BuPyOx) ligand

**DOI:** 10.3762/bjoc.9.187

**Published:** 2013-08-12

**Authors:** Hideki Shimizu, Jeffrey C Holder, Brian M Stoltz

**Affiliations:** 1 The Warren and Katharine Schlinger Laboratory for Chemistry and Chemical Engineering, Division of Chemistry and Chemical Engineering, California Institute of Technology, Pasadena, California 91125, USA; 2 Shionogi & Co., Ltd. 1-3, Kuise Terajima 2-chome Amagasaki, Hyogo 660-0813, Japan

**Keywords:** asymmetric catalysis, diamine ligands, optimization, synthesis

## Abstract

An efficient method for the synthesis of the (*S*)-4-(*tert*-butyl)-2-(pyridin-2-yl)-4,5-dihydrooxazole ((*S*)-*t-*BuPyOx) ligand has been developed. Inconsistent yields and tedious purification in known routes to (*S*)-*t-*BuPyOx suggested the need for an efficient, dependable, and scalable synthetic route. Furthermore, a route suitable for the synthesis of PyOx derivatives is desirable. Herein, we describe the development of a three-step route from inexpensive and commercially available picolinic acid. This short procedure is amenable to multi-gram scale synthesis and provides the target ligand in 64% overall yield.

## Introduction

**Figure 1:**
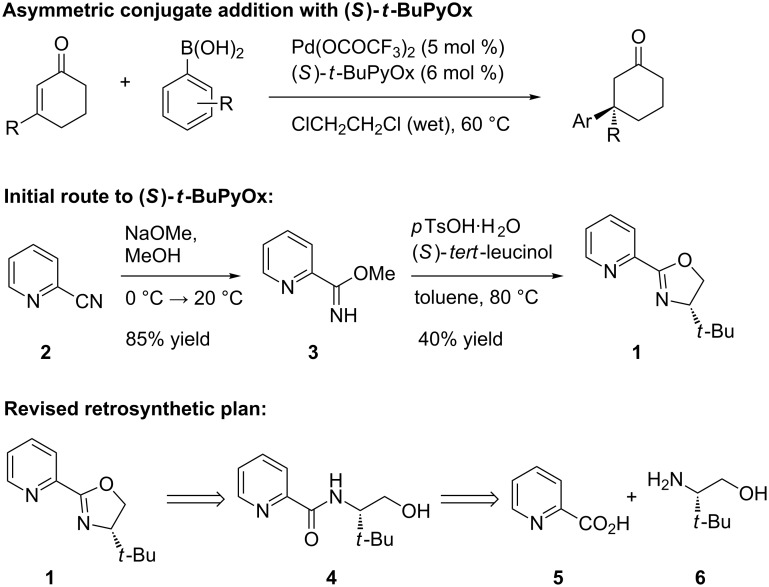
Initial PyOx synthesis and revised plan.

Pyridinooxazoline (PyOx) ligands represent a growing class of bidentate dinitrogen ligands used in asymmetric catalysis [1–23]. Recently, our laboratory reported the catalytic asymmetric conjugate addition of arylboronic acids to cyclic, β,β-disubstituted enones utilizing (*S*)-*t-*BuPyOx (**1**) as the chiral ligand (Figure 1) [24]. This robust reaction is insensitive to oxygen atmosphere, highly tolerant of water [25], and provides cyclic ketones bearing β-benzylic quaternary stereocenters in high yields and enantioselectivities. While the reaction itself proved to be amenable to multi-gram scale, the ligand is not yet commercially available and no reliable method for the large-scale synthesis of (*S*)-*t-*BuPyOx was known (a number of syntheses are known, including [26]). We sought to address this shortcoming by developing an efficient route starting from a cheap, commercially available precursor to pyridinooxazoline ligands. Herein, we report an efficient, highly scalable synthesis of (*S*)-*t-*BuPyOx.

## Results and Discussion

Initially, (*S*)-*t-*BuPyOx (**1**) was synthesized by methanolysis of 2-cyanopyridine (**2**) to afford methoxyimidate **3**, and subsequent acid-catalyzed cyclization to afford the (*S*)-*t-*BuPyOx ligand (Figure 1) [27]. We found the yields of this reaction sequence to be highly variable, and the purification by silica gel chromatography to be tedious. In the revised retrosynthesis, picolinic acid (**5**) was identified as a comparably priced, commonly available surrogate for cyanopyridine **2**. Amidation of (*S*)-*tert*-leucinol (**6**) and picolinic acid (**5**) would generate amide **4**, which upon cyclization would generate the ligand framework.

Initial efforts focused on the amidation reaction between (*S*)-*tert*-leucinol and picolinic acid (**5**) via acid chloride **7** (Table 1), which was generated in situ by treatment of acid **5** with a number of chlorinating agents. Oxalyl chloride (Table 1, entries 1,2) provided reasonable yields of amide **4**, however bis-acylation of (*S*)-*tert*-leucinol was observed as a common side product. Importantly, temperature control of this reaction (Table 1, entry 2) allowed the isolation of 75% of desired alcohol **4** in acceptable purity without the use of column chromatography. Use of diphenyl chlorophosphate (Table 1, entries 3,5,6) also resulted in noticeable quantities of over-acylation products, as well as the generation of a small amount of phosphorylation of amide **4**. These results encouraged us to explore alternative activation strategies to generate the desired amide bond. Adapting a procedure from Sigman, activation of acid **5** by treatment with isobutylchloroformate and *N*-methylmorpholine (anhydride **8**) facilitated the desired transformation with the highest overall yield, with amide **4** being isolated in 92% yield, albeit requiring column chromatography [28].

**Table 1:** Amidation reactions of picolinic acid.

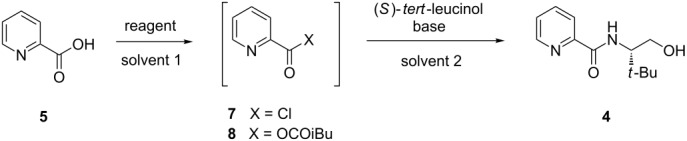						

entry	reagent	solvent 1/2	temp (°C)^a^	base	time (h)^b^	yield (%)^c^

1	(COCl)_2_	THF/THF	50	Et_3_N	1	55
2	(COCl)_2_	THF/THF	0 to rt	Et_3_N	7	75^d^
3	DPCP	THF/THF	0 to rt	Et_3_N	6	72
4	SOCl_2_	toluene/THF	rt	none	5	trace
5	DPCP	THF/THF	50	none	2	30
6	DPCP	THF/THF	0 to rt	Et_3_N	3	65^d^
7	iBuOCOCl, NMM	CH_2_Cl_2_/CH_2_Cl_2_	0 to rt	NMM	3	92

DPCP = diphenyl chlorophosphate, NMM = *N*-methylmorpholine. ^a^Temperature for second step; ^b^Time for second step; ^c^Isolated yield; ^d^Purification by flash chromatography not required.

Satisfied with our ability to generate amide **4** on gram-scale with good yield, we turned our attention to the completion of the synthesis. The cyclization of amide **4** to (*S*)-*t-*BuPyOx (**1**) proved more challenging than anticipated. Activation of alcohol **4** as mesylate **9** (Table 2, entries 1,2) and tosylate **10** (Table 2, entry 3) followed by in situ cyclization gave the desired product in low yield and incomplete conversion. This could potentially result from ligand hydrolysis under the given reaction conditions [29]. As an alternative to insitu cyclization of an activated intermediate, alcohol **4** was reacted with thionyl chloride (Table 2, entries 4–10) to yield chloride **11**, which was isolated as the hydrochloric acid salt and dried under vacuum. This compound proved to be bench stable and was spectroscopically unchanged after being left open to oxygen atmosphere and adventitious moisture for more than one week. Furthermore, chloride **11** proved to be a superior cyclization substrate. A series of bases were screened. Organic amine bases (Table 2, entries 4,5) and sodium hydride (Table 2, entry 6) provided inadequate conversion and low yields, whereas hydroxide and alkoxide bases proved superior (Table 2, entries 7–10). Finally, sodium methoxide was chosen to be optimal, as slower rates of hydrolysis of chloride **11** were observed when compared to the use of potassium hydroxide.

**Table 2:** Cyclization screen.

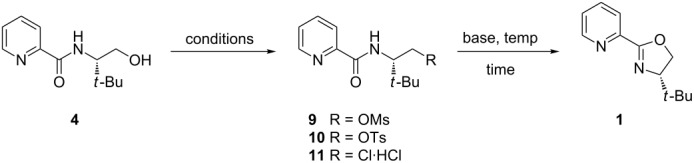						

entry	conditions	R	temp (°C)	base	time (h)	yield (%)^a^

1	MsCl, Et_3_N, CH_2_Cl_2_	OMs	0 to 40	Et_3_N	12	N.D.^b^
2	MsCl, Et_3_N, ClCH_2_CH_2_Cl	OMs	0 to 80	Et_3_N	12	N.D.^b^
3	TsCl, DMAP, Et_3_N, ClCH_2_CH_2_Cl	OTs	0 to 80	Et_3_N	12	N.D.^b^
4	SOCl_2_	Cl^c^	rt	DABCO	18	38
5	SOCl_2_	Cl^c^	50	DBU	12	59
6	SOCl_2_	Cl^c^	0 to 50	NaH, THF	18	60
7	SOCl_2_	Cl^c^	50	5% KOH/EtOH	11	58
8	SOCl_2_	Cl^c^	50	5% KOH/MeOH	11	62
9	SOCl_2_	Cl^c^	50	25% NaOMe/MeOH	10	71
10	SOCl_2_	Cl^c^	50	25% NaOMe/MeOH	3	72

MsCl = methanesulfonyl chloride, TsCl = 4-toluenesulfonyl chloride, DMAP = 4-dimethylaminopyridine, DABCO = 1,4-diazabicyclo[2.2.2]octane, DBU = 1,8-diazabicyclo[5.4.0]undec-7-ene. ^a^Isolated yield; ^b^Incomplete conversion; ^c^Intermediate **11** isolated as HCl salt and dried under high vacuum before use in cyclization reactions.

Attempts to purify ligand **1** via salt formation failed due to instability of the generated products [30]. Purification by silica gel chromatography also proved challenging as up to 10% of crude ligand **1** was observed to decompose, even with the addition of triethylamine to the eluent. Finally, the use of neutral silica gel (American International Chemical ZEOprep ECO silica gel, 40–63 micron, $18/kg) allowed isolation of ligand **1** in high purity and with no observed decomposition.

## Conclusion

**Figure 2:**
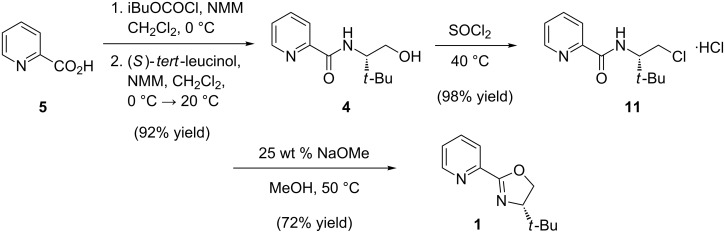
Scale-up synthesis of (*S*)-*t-*BuPyOx.

In conclusion, we have developed a concise, highly efficient and scalable synthesis of the chiral ligand (*S*)-*t-*BuPyOx (**1**) (Figure 2). Efforts to further refine the synthesis by telescoping the procedure and removing chromatographic purifications are currently underway.

## Experimental


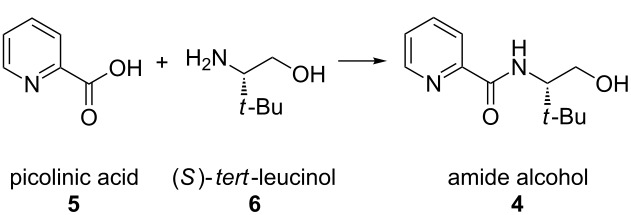


**(*S*)-*N*-(1-hydroxy-3,3-dimethylbutan-2-yl)picolinamide (4):** To a 200 mL round bottom flask was added picolinic acid (2.46 g, 20.0 mmol, 1.00 equiv), 50 mL CH_2_Cl_2_, and *N*-methylmorpholine (3.03 g, 30.0 mmol, 1.50 equiv). The reaction mixture was cooled to 0 °C in an ice bath and isobutyl chloroformate (3.14 g, 23.0 mmol, 1.15 equiv) was added dropwise over 30 min. Following complete addition, the reaction mixture was stirred for 30 min at 0 °C. In a separate flask, (*S*)-*tert*-leucinol (2.58 g, 22.0 mmol, 1.10 equiv) was dissolved in CH_2_Cl_2_ (25 mL), and *N*-methylmorpholine (2.43 g, 24.0 mmol, 1.20 equiv) was added. This solution was transferred dropwise over the course of 1 h to the cooled reaction mixture using a syringe pump. The cooling bath was removed and the reaction mixture was allowed to warm to room temperature and stirred for 2 h. The mixture was quenched with an aqueous solution of NH_4_Cl (10 g in 50 mL H_2_O) and the aqueous phase was extracted with CH_2_Cl_2_ (20 mL). The combined organic phase was dried over Na_2_SO_4_ (5 g), filtered, and concentrated under reduced pressure. The residue was purified with flash silica gel column chromatography (4:1 hexanes/acetone) to afford amide alcohol **4** as a white solid (4.10 g, 92% yield). *R*_f_ 0.32 with 3:2 hexanes/acetone; mp 79.6–79.9 °C; ^1^H NMR (500 MHz, CDCl_3_) δ 8.56 (ddd, *J* = 4.8, 1.8, 0.9 Hz, 1H), 8.32 (br d, *J* = 8.9 Hz, -NH), 8.19 (dt, *J* = 7.8, 1.1 Hz, 1H), 7.85 (td, *J* = 7.7, 1.7 Hz, 1H), 7.43 (ddd, *J* = 7.6, 4.8, 1.2 Hz, 1H), 4.02–3.96 (m, 2H), 3.69 (m, 1H), 2.72 (br t, *J* = 6.5 Hz, -OH), 1.05 (s, 9H); ^13^C NMR (125 MHz, CDCl_3_) δ 165.6, 149.7, 148.2, 137.6, 126.4, 122.6, 63.7, 60.6, 33.9, 27.1; IR (neat film, NaCl): 3375, 2962, 1669, 1591, 1570, 1528, 1465, 1434, 1366, 1289, 1244, 1088, 1053, 998 cm^−1^; HRMS (MultiMode ESI/APCI) *m*/*z*: [M + H]^+^ calcd for C_12_H_19_N_2_O_2_, 223.1447; found, 223.1448; [α]^25^_D_ −8.7 (*c* 1.17, CHCl_3_, >99% ee).


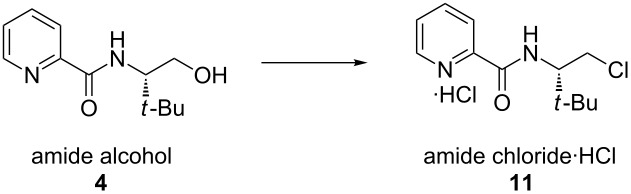


**(*S*)-*N*-(1-chloro-3,3-dimethylbutan-2-yl)picolinamide hydrochloride (11):** A 500 mL 3-neck round bottom flask was charged with a stir bar, amide alcohol **4** (8.89 g, 40.0 mmol, 1.00 equiv) and toluene (140 mL). The resulting clear solution was warmed to 60 °C. In a separate flask, SOCl_2_ (9.25 g, 80.0 mmol, 2.00 equiv) was diluted with toluene (20 mL). This solution was transferred slowly, dropwise, over 20 min to the vigorously stirring reaction mixture at 60 °C. The reaction mixture was stirred at 60 °C for 4 h, at which time the slurry was cooled to ambient temperature, concentrated on a rotary evaporator under reduced pressure (40 °C, 40 mmHg), and dried under vacuum (0.15 mmHg) to give a white powder of amide chloride hydrochloric salt **11** (10.80 g, 98% yield). This material was used in the following step without purification. ^1^H NMR (500 MHz, DMSO-*d*_6_) δ 8.70 (ddd, *J* = 4.8, 2.0, 1.0 Hz, 1H), 8.66 (br d, *J* = 9.9 Hz, -NH), 8.10 (dt, *J* = 8.0, 1.0 Hz, 1H), 8.06 (td, *J* = 7.5, 1.4 Hz, 1H), 7.66 (ddd, *J* = 7.4, 4.8, 1.4 Hz, 1H), 4.08 (td, *J* = 9.9, 3.7 Hz, 1H), 3.97–3.90 (m, 2H), 0.93 (s, 9H); ^13^C NMR (125 MHz, DMSO-*d*_6_) δ 163.6, 149.0, 147.8, 138.1, 126.5, 122.0, 59.0, 44.9, 35.0, 26.3; IR (neat film, NaCl): 3368, 2963, 1680, 1520, 1465, 1434, 1369, 1285, 1239, 1087, 998 cm^−1^; HRMS (MultiMode ESI/APCI) *m*/*z*: [M + H]^+^ calcd for C_12_H_18_ClN_2_O, 241.1108; found, 241.1092; [α]^25^_D_ +39.4 (*c* 0.96, MeOH, >99% ee).


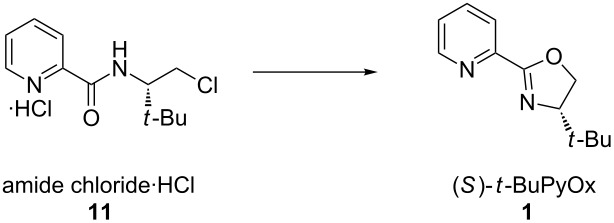


**(*S*)-4-(*tert*-butyl)-2-(pyridin-2-yl)-4,5-dihydrooxazole (1):** A 500 mL 3-neck round bottom flask was charged with a stir bar, amide chloride hydrochloric acid salt **11** (10.26 g, 37.0 mmol, 1.00 equiv) and MeOH (100 mL). To the clear solution was added powdered NaOMe (9.99 g, 185.0 mmol, 5.00 equiv), and the resulting mixture was heated to 55 °C in an oil bath. The slurry was stirred for 3 h until the free amide chloride was fully consumed, according to TLC analysis (4:1 hexanes/acetone). After removing the oil bath, toluene (100 mL) was added and the mixture was concentrated on a rotary evaporator (40 °C, 60 mmHg) to remove MeOH. The residual mixture was extracted with H_2_O (100 mL) and the aqueous phase was back extracted with toluene (40 mL × 2). The combined organic extracts were dried over Na_2_SO_4_ (10 g), filtered, and concentrated under reduced pressure. The residue was purified by flash column chromatography using American International Chemical ZEOprep® 60 ECO 40-63 micron silica gel (4:1 hexanes/acetone) to yield (*S*)-*t*-BuPyOx (**1**) as a white solid (5.44 g, 72% yield). *R*_f_ 0.44 with 3:2 hexanes/acetone; mp 70.2–71.0 °C; ^1^H NMR (500 MHz, CDCl_3_) δ 8.71 (ddd, *J* = 4.8, 1.8, 0.9 Hz, 1H), 8.08 (dt, *J* = 7.9, 1.1 Hz, 1H), 7.77 (dt, *J* = 7.7, 1.7 Hz, 1H), 7.37 (ddd, *J* = 7.0, 4.5, 1.0 Hz, 1H), 4.45 (dd, *J* = 10.2, 8.7 Hz, 1H), 4.31 (t, *J* = 8.5 Hz, 1H), 4.12 (dd, *J* = 10.2, 8.5 Hz, 1H), 0.98 (s, 9H); ^13^C NMR (125 MHz, CDCl_3_) δ 162.4, 149.6, 147.0, 136.5, 125.4, 124.0, 76.5, 69.3, 34.0, 26.0; IR (neat film, NaCl): 2981, 2960, 2863, 1641, 1587, 1466, 1442, 1358, 1273, 1097, 1038, 968 cm^−1^; HRMS (MultiMode ESI/APCI) *m*/*z*: [M + H]^+^ calcd for C_12_H_17_ON_2_, 205.1335; found, 205.1327; [α]^25^_D_ −90.5 (*c* 1.15, CHCl_3_, >99% ee).

## Supporting Information

Materials and methods, auxiliary experimental details, and relevant NMR spectra are provided.

File 1 Experimental details.
