# Supplementary material for: A scalable synthesis of the (S)-4-(tert-butyl)-2-(pyridin-2-yl)-4,5-dihydrooxazole ((S)-t-BuPyOx) ligand
Source: Beilstein J Org Chem. 2013 Aug 12;9:1637–42. doi: 10.3762/bjoc.9.187 (PMC3778391; doi:10.3762/bjoc.9.187)
Supplement: File 1 — Experimental details. [file Beilstein_J_Org_Chem-09-1637-s001.pdf]

Supporting Information

for

A scalable synthesis of the (*S*)-4-(*tert*-butyl)-2-(pyridin-2-yl)-4,5-dihydrooxazole ((*S*)-*t*-BuPyOx) ligand

Hideki Shimizu[1,2], Jeffrey C. Holder[1] and Brian M. Stoltz*[1]

Address: [1]The Warren and Katharine Schlinger Laboratory for Chemistry and Chemical Engineering, Division of Chemistry and Chemical Engineering, California Institute of Technology, Pasadena, California 91125, USA and [2]Shionogi & Co., Ltd. 1-3, Kuise Terajima 2-chome Amagasaki, Hyogo 660-0813, Japan

Email: Brian M. Stoltz* - stoltz@caltech.edu

*Corresponding Author

Experimental details

Table of contents:

Materials and methods S2

Experimental procedures S3

^{1}H and ^{13}C NMR spectra S7

Materials and methods

All reactions were run under a nitrogen atmosphere. Solvents and reagents were obtained by commercial sources and used without further purification. Thin layer chromatography (TLC) was visualized by UV fluorescence quenching, and *p*-anisaldehyde staining. American International Chemical ZEOprep® 60 ECO 40-63 micron silica gel was used for flash chromatography. Analytical chiral SFC was performed utilizing an OB-H column (4.6 mm × 25 cm) with visualization at 254 nm and flow rate of 5.0 mL/min, unless otherwise stated. ^{1}H and ^{13}C NMR spectra were recorded at 500 MHz and 125 MHz, respectively. Data for ^{1}H NMR spectra are referenced to the centerline of $CHCl_3$ (δ 7.26) as the internal standard and are reported in terms of chemical shift relative to Me_4Si (δ 0.00). Data for ^{13}C NMR spectra are referenced to the centerline of $CDCl_3$ (δ 77.16) and are reported in terms of chemical shift relative to Me_4Si (δ 0.00). Infrared spectra are reported in frequency of absorption (cm^{-1}).

Experimental procedures

(*S*)-*tert*-Leucinol (**6**)

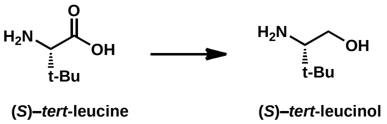

This procedure was adapted from: Krout, M. R.; Mohr, J. T.; Stoltz, B. M. *Org. Synth*. **2009,** *86*, 181. A 1000 mL separable flask equipped with a three-pitched curved blade, an internal thermometer, and a reflux condenser equipped with a two-tap Schlenk adapter connected to a bubbler and a nitrogen/vacuum manifold was assembled hot and cooled under a steam of N_2. The flask was charged with (*S*)-*tert*-leucine (15.08 g, 115.0 mmol, 1.00 equv, 99% ee) and THF (360 mL) under a positive pressure of nitrogen. The resulting slurry was cooled to 0 ºC in a dry ice-acetone bath and $NaBH_4$ (10.44 g, 276.0 mmol, 2.40 equiv) was added in one portion. A solution of I_2 (29.19 g, 115.0 mmol, 1.00 equiv) in THF (50 mL) was transferred dropwise over 2 hours by using a syringe pump. After the addition was complete, the cooling bath and the thermometer were removed and replaced by a condenser and the reaction was heat to reflux (80 °C oil bath). After 20 h the reaction was allowed to cool to ambient temperature and methanol (150 mL) was added slowly, resulting in an almost clear solution. After stirring for 30 min the solution was quantitatively transferred to a 1000 mL round bottom flask with MeOH (100 mL) and concentrated on a rotary evaporator under reduced pressure (40 °C) to a white semi-solid. The resulting material was dissolved in 20 wt % aqueous KOH (250 g) and stirred for 12 h at ambient temperature. The aqueous phase was extracted with CH_2Cl_2 (5 × 180 mL) and the combined organic extracts were dried over Na_2SO_4 (ca. 25 g), filtered, and concentrated on a rotary evaporator under reduced pressure

(40 °C) and dried under vacuum to give crude (*S*)-*tert*-leucinol (14.00 g, quantitative yield) as a colorless oil. This material was used in the following step without further purification.

Attempts to Purify *t*-BuPyOx by Salt Formation

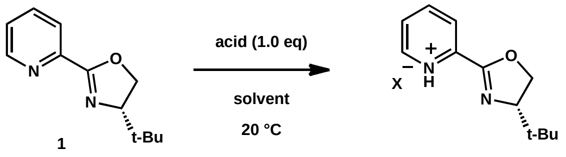

Table 7. Screening of salt formation with various acids.

Entry	Acid	Scale	Solvent	Time	Result
1	maleic acid	5.1 mg	THF	8 h	peak shifted (mixture)
2	fumaric acid	5.1 mg	THF	8 h	not formed
3	malonic acid	5.1 mg	THF	8 h	peak shifted (mixture)
4	DL-malic acid	5.1 mg	THF	8 h	not formed
5	2-ethylhexanoic acid	5.1 mg	THF	8 h	not formed
6	HCl in ether	10 mg	ether	12 h	decomposed
7	HBF4 etherate	10 mg	ether	12 h	salt formed*

* PyOX was partly decomposed to amide alcohol.

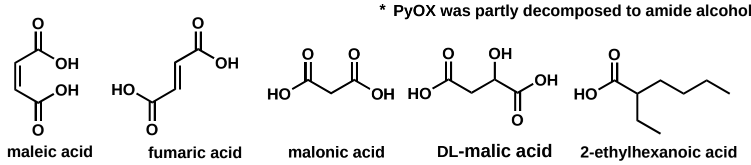

While purification via formation of the HBF_4 salt is successful, the salt is unstable in open air and decomposes to the amide alcohol **4**. Although salt formation is perhaps useful in the purification of the crude reaction mixture of **1**, further studies were suspended due to the instability of salt.

Alternative Route to (*S*)–*t*-BuPyOx

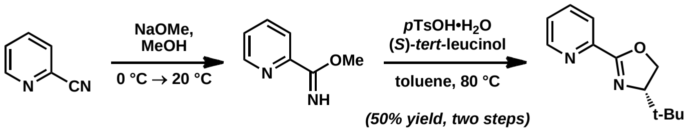

This route is amenable to smaller batches of ligand synthesis. Adapted from Brunner, H.; Obermann. U. *Chem. Ber.* **1989,** *122,* 499–507.

A flame-dried round bottom flask was charged with a stir bar and MeOH (110 mL). Sodium metal ingot (295 mg, 12.8 mmol, 0.1 equiv) was cut with a razor into small pieces, washed in a beaker of hexanes, and added in five portions over 5 min. The reaction mixture was stirred vigorously until no sodium metal remained, at which time it was cooled to 0 °C in an ice/water bath. Subsequently, 2-cyanopyridine (13.0 g, 125 mmol, 1.0 equiv) was added drop wise, and the clear, colorless reaction mixture was allowed to warm to ambient temperature and stir. When all the starting material was consumed as indicated by TLC analysis (1:1 EtOAc/hexanes, *p*-anisaldehyde stain), the reaction was cooled to 0 °C in an ice/water bath and quenched dropwise with glacial AcOH (1 mL). The crude reaction mixture was concentrated in vacuo, redissolved in CH_2Cl_2 (100 mL) and washed with brine (2 × 50 mL). The organic phase was dried ($MgSO_4$), concentrated in vacuo, and dried under high vacuum for 1 h. The resulting crude methoxyimidate (light yellow oil) was suitable for use in the next step without further purification.

To a flame-dried round bottom flask charged with a stir bar was added crude methoxyimidate (2.55 g, 18.7 mmol, 1.0 equiv), (*S*)-*tert*-leucinol (2.10 g, 17.9 mmol, 0.96 equiv), toluene (100 mL), and *p*-TsOH·H_2O (167 mg, 0.88 mmol, 5 mol %). The mixture was stirred at 80 °C in an oil bath for 3 h, at which time the starting material was

consumed as indicated by TLC analysis (1:4 acetone/hexanes, *p*-anisaldehyde stain). The reaction was cooled to ambient temperature and quenched with sat. $NaHCO_3$ (60 mL). The reaction was partitioned with EtOAc and water, and the aqueous phase was extracted with EtOAc (3 × 50 mL). The combined organic extracts were washed with water (2 × 50 mL), brine (1 × 25 mL), dried ($MgSO_4$) and concentrated in vacuo. The crude mixture was purified by flash column chromatography using American International Chemical ZEOprep® 60 ECO 40-63 micron silica gel (1:4 acetone/hexanes) to afford 1.85 g (9.06 mmol, 50%) (*S*)-*t*-BuPyOx as an off-white solid. Characterization data matches the data previously reported in this document.

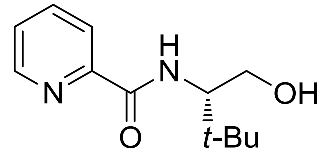

4

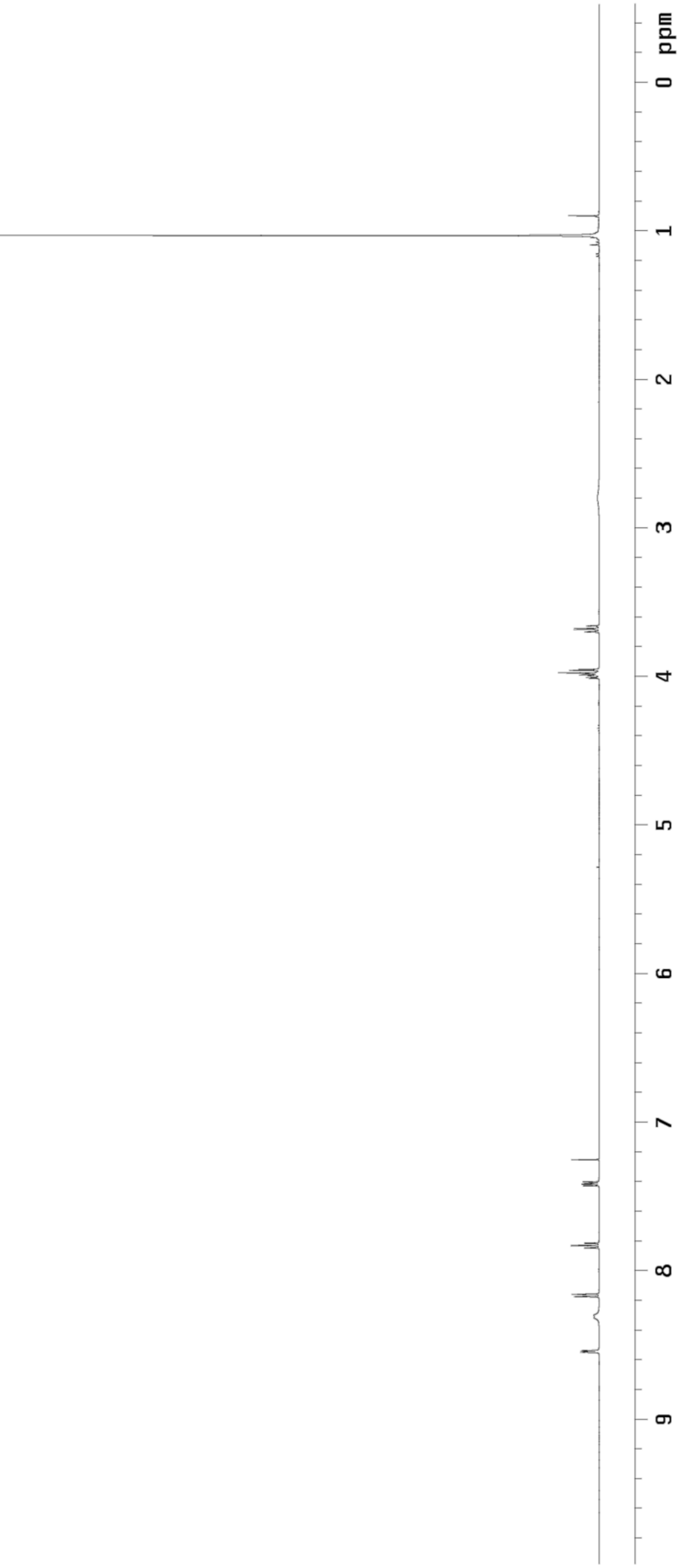

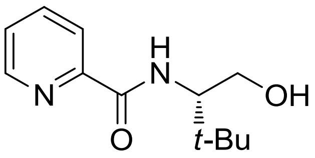

4

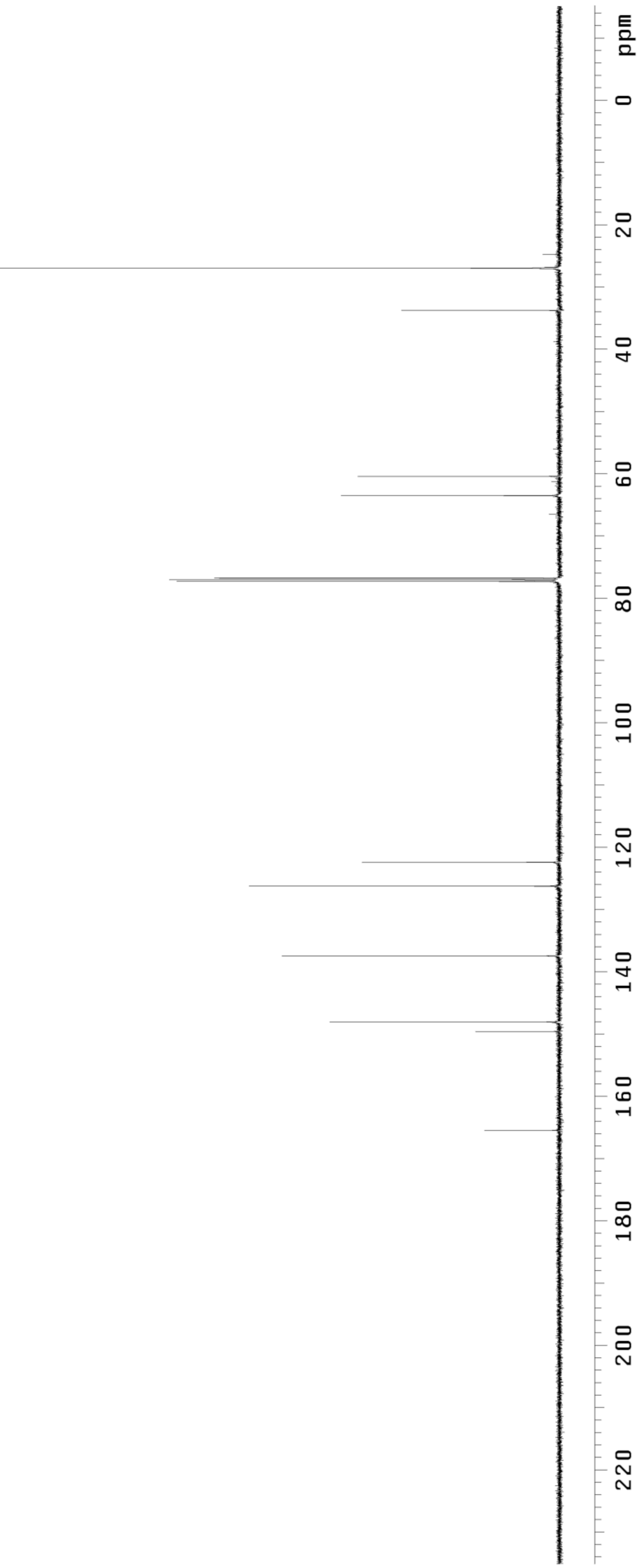

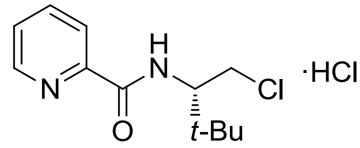

11

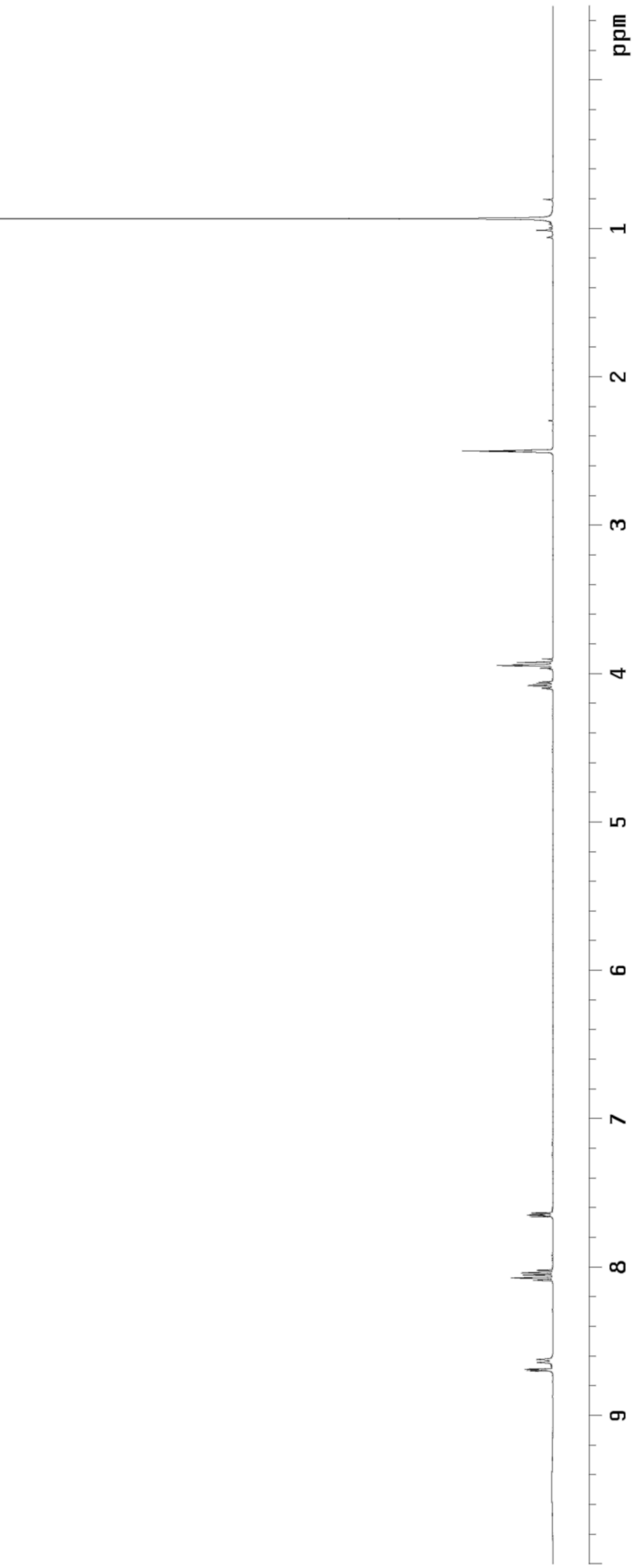

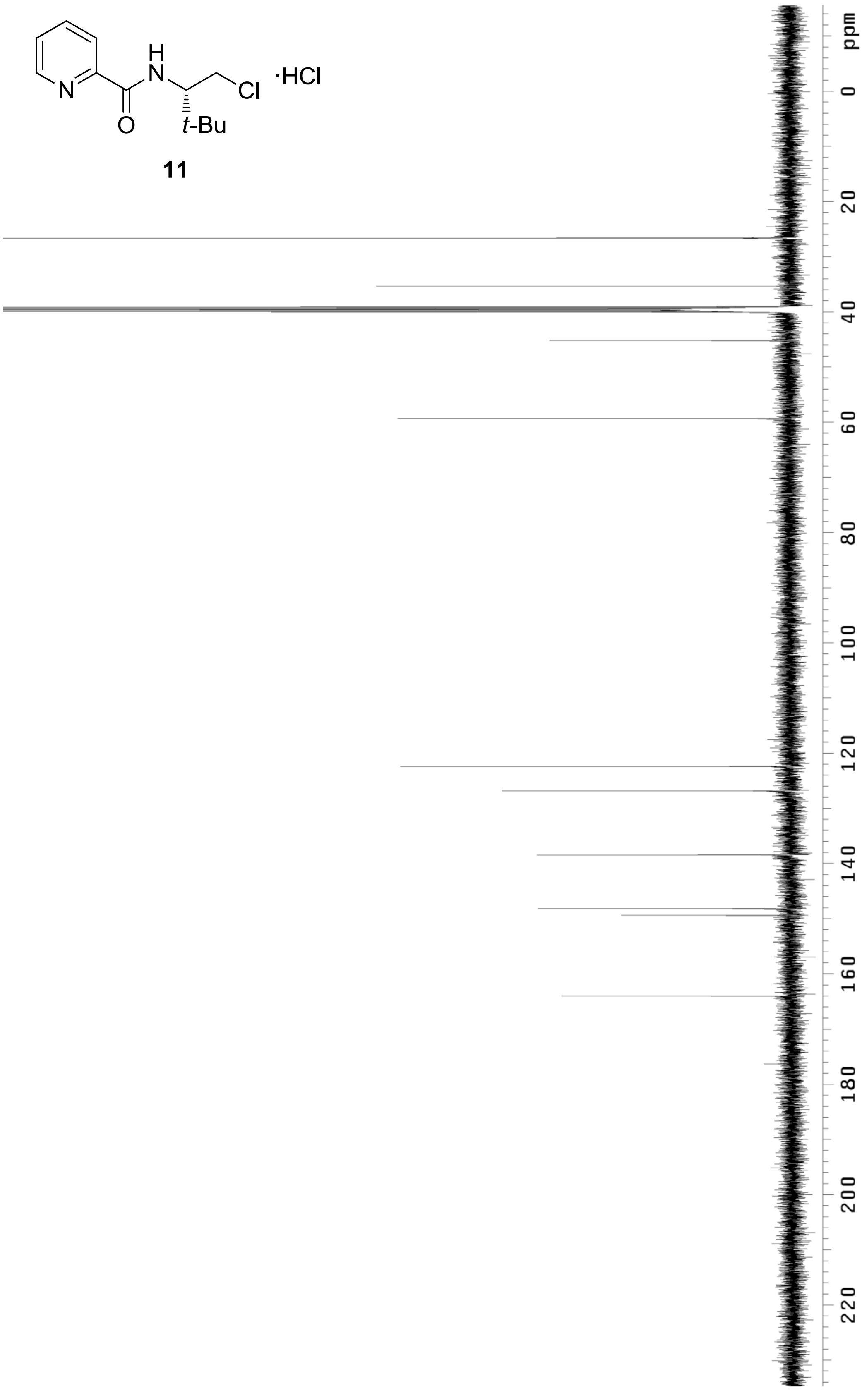

N
H
N
O
Cl
·HCl
t-Bu
11
ppm
0
20
40
60
80
100
120
140
160
180
200
220

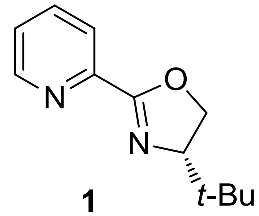

1

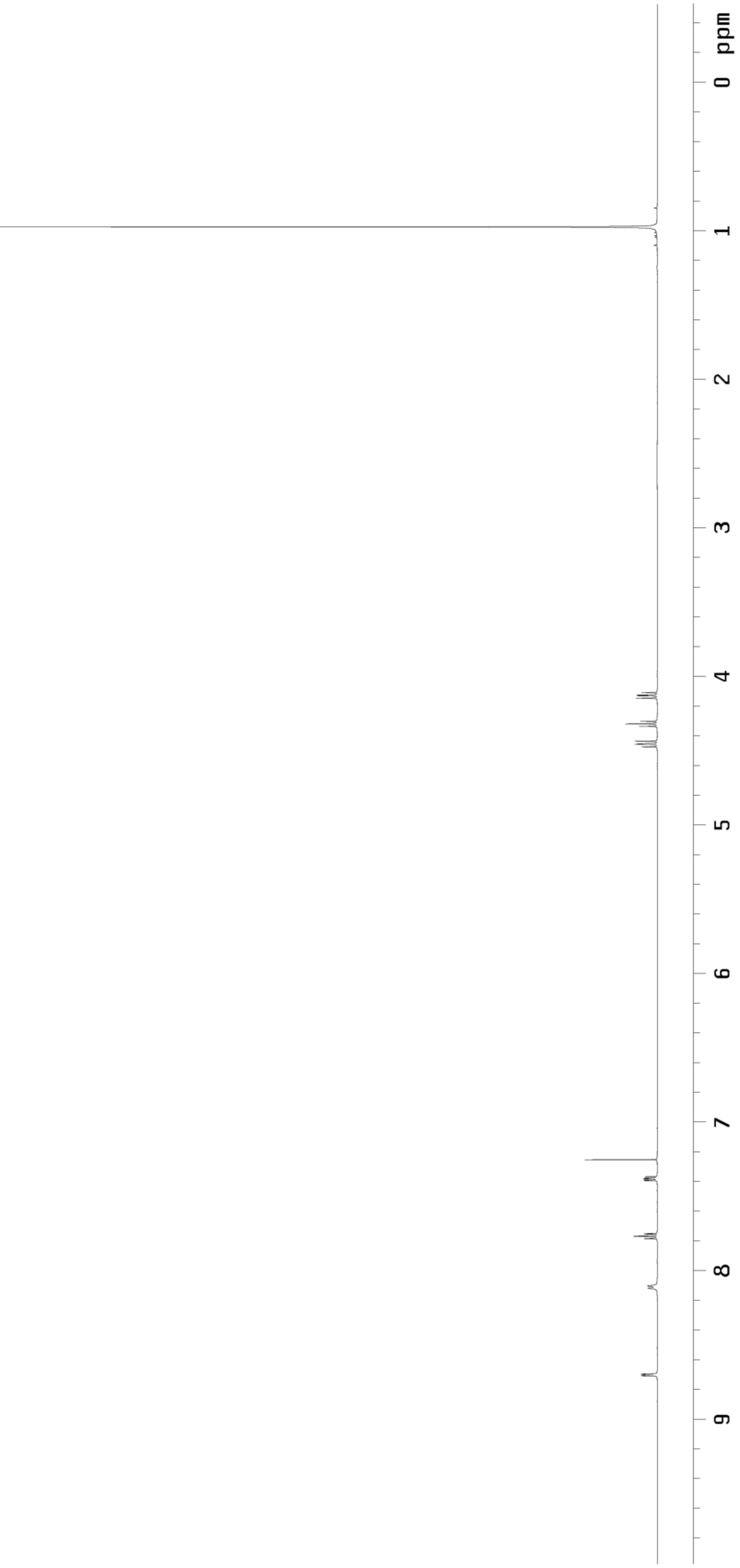

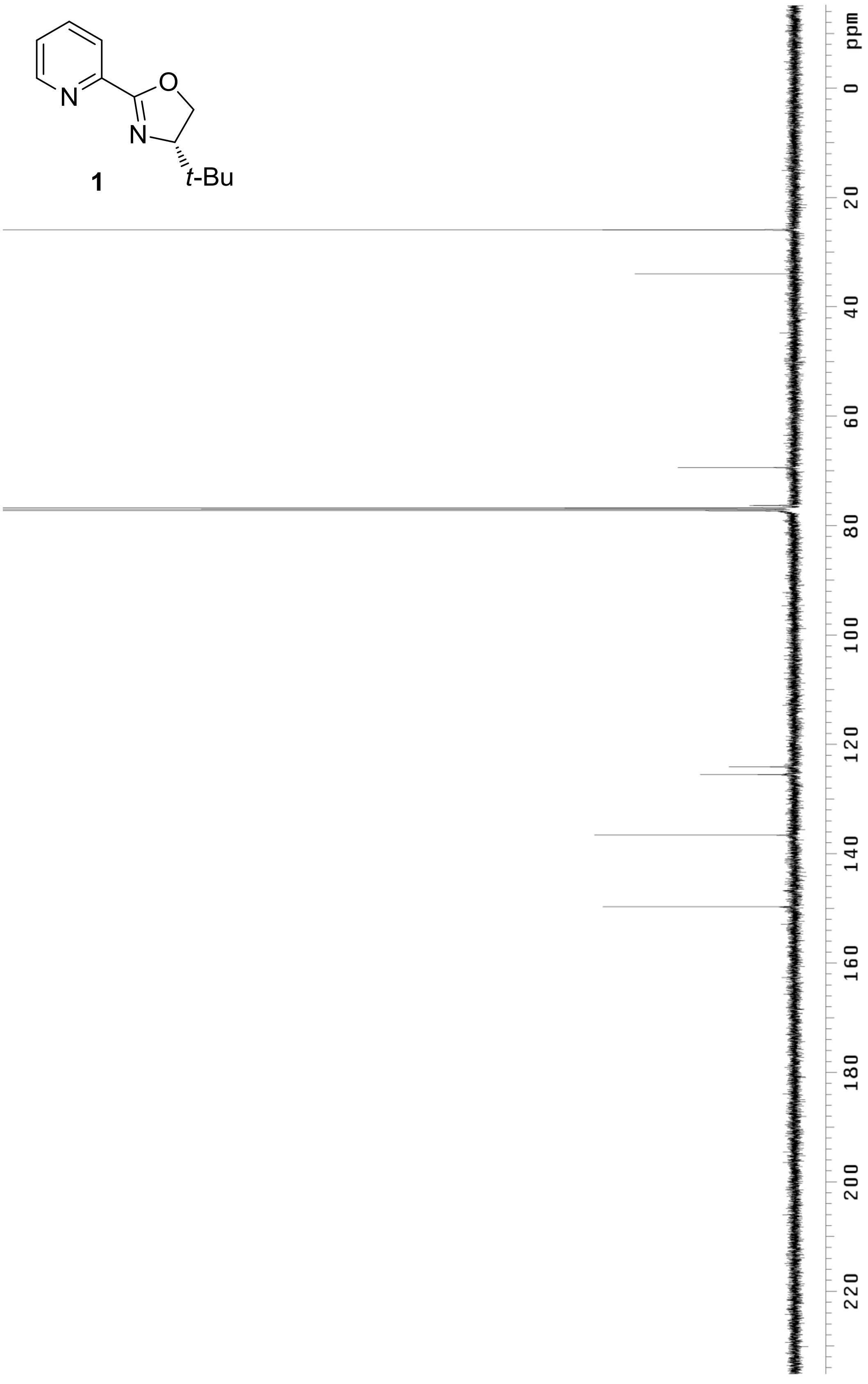
N
O
N
t-Bu
1
ppm
0
20
40
60
80
100
120
140
160
180
200
220
